# Supplementary material for: EPEE: towards efficient and effective foundation models in biomedicine
Source: Npj Health Syst. 2026 May 12;3:30. doi: 10.1038/s44401-026-00083-2 (PMC13167461; doi:10.1038/s44401-026-00083-2)
Supplement: Supplementary file 1 — Supplementary information [file 44401_2026_83_MOESM1_ESM.pdf]

Theoretical Computational Complexity (FLOPs) with/without Early Exit

We count one multiply–add as 2 FLOPs. Let B be batch size, L sequence length, d hidden size, d_{ff} FFN size (typically $d_{ff} = 4d$), and C the number of classes. We ignore LayerNorm/residual/softmax as lower-order terms dominated by GEMMs.

Backbone FLOPs per Transformer layer. Self-attention:

$$F_{\text{attn}} = (6BLd^2)_{\text{QKV}} + (2BL^2d)_{QK^\top} + (2BL^2d)_{AV} + (2BLd^2)_{\text{out}} = 8BLd^2 + 4BL^2d.$$

FFN ($d \to d_{ff} \to d$):

$$F_{\text{ffn}} = 2BLdd_{ff} + 2BLd_{ff}d = 4BLdd_{ff}.$$

Thus,

$$F_{\text{layer}} = F_{\text{attn}} + F_{\text{ffn}} = 4BL^2d + 8BLd^2 + 4BLdd_{ff} \quad (\text{if } d_{ff} = 4d: \ F_{\text{layer}} \approx 4BL^2d + 24BLd^2).$$

Classifier head FLOPs (treated as a linear layer). For a linear classifier $d \to C$ applied on a pooled/CLS representation:

$$F_{\text{head}} = 2BdC$$

FLOPs without early exit (full inference). For an M-layer backbone:

$$F_{\text{full}} = M \cdot F_{\text{layer}} + F_{\text{head}}$$

FLOPs with early exit. If an input exits at layer $m \in \{1, \dots, M\}$ (executing the corresponding exit head once):

$$F_{\text{exit}}(m) = m \cdot F_{\text{layer}} + F_{\text{head}}$$

For a dataset of N samples with exit layers $\{m_i\}_{i=1}^N$, the average FLOPs are

$$\overline{F}_{\text{exit}} = \frac{1}{N} \sum_{i=1}^{N} (m_i \cdot F_{\text{layer}} + F_{\text{head}}) = \left(\frac{1}{N} \sum_{i=1}^{N} m_i \right) F_{\text{layer}} + F_{\text{head}}$$

Theoretical FLOPs reduction

$$\Delta F = F_{\text{full}} - \overline{F}_{\text{exit}} = \left(M - \frac{1}{N} \sum_{i=1}^{N} m_i \right) F_{\text{layer}}.$$
